# Integrated network toxicology and experimental validation identify VCAM1 as a candidate molecular link between pyrethroid exposure and keloid

**DOI:** 10.1371/journal.pone.0362474

**Published:** 2026-11-10

**Authors:** Zun Wang, Chen Yang, Wencai Wang, Zijian Zhang, Zhonghua Sun, Zhiyong Diao

**Affiliations:** 1 The Second Affiliated Hospital of Harbin Medical University, Harbin, China; 2 Shenyang Fifth People’s Hospital, Shenyang, China; 3 The First Affiliated Hospital of Harbin Medical University, Harbin, China; 4 Medical School of Nanjing University, Nanjing, China; Shiraz University, IRAN, ISLAMIC REPUBLIC OF

## Abstract

**Background:**

Keloids are fibroproliferative skin lesions characterized by excessive extracellular matrix deposition. The potential molecular links between pyrethroid exposure and keloid-associated alterations remain unclear.

**Methods:**

We integrated network toxicology, machine learning, single-cell transcriptomics, virtual gene perturbation, molecular docking, molecular dynamics simulation, external transcriptomic validation, and qRT-PCR to investigate candidate molecular links between pyrethroid exposure and keloid.

**Results:**

A total of 127 shared candidate targets between pyrethroid insecticides and keloid were identified, mainly enriched in MAPK, PI3K–Akt, and FoxO signaling pathways. Three machine-learning algorithms prioritized VCAM1 and FGFR1. In GSE145725, both genes showed significant differential expression and high dataset-level discriminatory performance. In the integrated external validation cohort generated from GSE90051 and GSE158395, VCAM1 remained significantly upregulated and showed good discriminatory performance (AUC = 0.869), whereas FGFR1 showed weaker external support (AUC = 0.677) without significant differential expression. Molecular docking and 100-ns molecular dynamics simulation supported the structural plausibility of selected pyrethroid–target interactions, particularly theta-cypermethrin with VCAM1. Single-cell analysis characterized the cell-type-specific distribution of candidate genes, while virtual gene perturbation suggested a putative VCAM1–SOCS3 regulatory relationship. qRT-PCR further showed that 20 μM cypermethrin exposure for 24 h significantly increased VCAM1 mRNA expression in KEL FIB cells.

**Conclusion:**

VCAM1 emerged as the most consistently supported candidate linking cypermethrin exposure with keloid-associated molecular alterations, while the predicted VCAM1–SOCS3 relationship requires further mechanistic validation.

## 1 Introduction

Keloids are persistent fibroinflammatory skin conditions defined by abnormal wound healing, involving excessive deposition of extracellular matrix and ongoing fibrotic remodeling [1,2]. Clinically, they exhibit high recurrence rates and substantial resistance to current therapies [3]. Epidemiological studies suggest that keloids are more prevalent in equatorial regions (e.g., Africa, Asia, Japan, and Latin America), with reported incidence estimates of approximately 3.5% in Cameroon and 0.1% in Japan, whereas incidence appears substantially lower in Europe and North America (both <0.1%) [4]. Keloids typically arise following cutaneous injury or inflammatory insults—such as insect bites, varicella, or herpes zoster—and are frequently accompanied by pruritus and pain [5]. Current evidence indicates that sustained inflammation, oxidative stress, and immune-microenvironment dysregulation act in concert to promote aberrant fibroblast activation, constituting a key pathological basis for keloid initiation and progression [6].

Pyrethroid insecticides are widely used, high-efficacy, broad-spectrum agents employed in household and agricultural settings for pest control [7,8]. As prevalent environmental and occupational toxicants, pyrethroid insecticides can bioaccumulate following repeated exposure and contribute to disease development by inducing oxidative stress, perturbing immune regulation, and disrupting endocrine homeostasis [9,10]. Evidence suggests that pyrethroid pesticides can directly activate microglia via interactions with microglial voltage-gated sodium channels, thereby modulating neuroinflammatory signaling and potentially contributing to the pathogenesis of neurodegenerative diseases [11]. In addition, pyrethroids may affect the development and occurrence of various respiratory diseases through multiple targets, thereby regulating metabolic disorders, endocrine disturbances and inflammation [12]. However, evidence linking pesticide exposure to keloid risk—and the underlying biological mechanisms—remains sparse and fragmented, underscoring the need for systematic, integrative investigation.

Network toxicology links toxicant-associated targets with disease-related genes to identify perturbed biological pathways and generate testable molecular hypotheses connecting environmental exposures with disease [13–16]. In this study, we integrated network toxicology, machine learning, molecular docking [17], molecular dynamics simulation, single-cell transcriptomics [18], virtual gene perturbation, external transcriptomic validation, and qRT-PCR to investigate potential molecular links between pyrethroid exposure and keloid-associated alterations. Network toxicology combined with machine-learning approaches [19] prioritized VCAM1 and FGFR1 as candidate genes, and their dataset-level discriminatory performance was subsequently evaluated using receiver operating characteristic (ROC) analysis. Independent transcriptomic validation further assessed the reproducibility of their expression patterns. ssGSEA-based immune infiltration analysis and Mendelian randomization were used to explore associations between VCAM1 and immune-cell traits, whereas single-cell transcriptomic profiling characterized the cell-type-specific distribution of VCAM1 and FGFR1 across keloid cellular populations. Molecular docking and molecular dynamics simulations provided supportive computational evidence for selected pyrethroid–target interactions. Virtual gene perturbation further suggested a putative regulatory relationship between VCAM1 and SOCS3. In addition, qRT-PCR showed that cypermethrin exposure increased VCAM1 mRNA expression in keloid-derived fibroblasts. Collectively, these findings identify VCAM1 as a prioritized candidate potentially linking pyrethroid exposure with keloid-associated molecular alterations, while the predicted VCAM1–SOCS3 relationship remains hypothesis-generating and requires further mechanistic validation.

## 2 Materials and methods

### 2.1 Screening of pyrethroid insecticides -associated targets

Representative insecticidal compounds were selected based on prior literature, including cyphenothrin, dimefluthrin, imiprothrin, prallethrin, Theta-cypermethrin, and transfluthrin. Canonical SMILES strings were retrieved from the PubChem database, and corresponding 3D structures were downloaded. Acute toxicity and ADMET-related properties were subsequently predicted using ProTox-3.0 and ADMETlab 3.0.

We retrieved putative gene targets for the six insecticides from three complementary databases (SEA, SwissTargetPrediction, and TargetNet). All targets were aggregated and de-duplicated to generate a non-redundant target set. Target identifiers were subsequently standardized and mapped to official gene symbols using the UniProt database.

### 2.2 Screening of Keloids -associated targets

To comprehensively capture keloid-associated genes, we queried three curated resources (GeneCards, TTD, and OMIM) to collect reported targets. In parallel, we performed differential expression analysis using the GSE145725 [20] dataset to identify keloid-related DEGs. Genes from these sources were subsequently integrated and de-duplicated to generate a consolidated keloid-associated gene set.

### 2.3 Pathway enrichment analysis and protein–protein interaction (PPI) network construction of shared genes

The keloid-associated gene set was intersected with the pyrethroid insecticide–related targets to obtain shared candidate genes. Kyoto Encyclopedia of Genes and Genomes (KEGG) and Gene Ontology (GO) enrichment analyses were then conducted to characterize the biological processes and signaling pathways represented by these shared genes. Finally, the shared genes were submitted to the STRING database to construct a PPI network and delineate their interaction architecture.

### 2.4 Candidate target prioritization using three machine-learning algorithms

To prioritize core targets associated with pyrethroid-related keloid pathogenesis, we employed three complementary machine-learning approaches—random forest, support vector machine–recursive feature elimination (SVM-RFE), and LASSO regression–based feature selection—to identify informative features. Candidate genes consistently selected across all three methods were intersected and defined as the core target set.

### 2.5 ROC curve analysis to assess the diagnostic performance of candidate genes

GSE145725 was used as the training dataset for candidate gene identification and model development. For external validation, GSE90051 [21] and GSE158395 [22] were independently preprocessed and normalized according to their respective platform characteristics. Genes shared between the two datasets were retained, and expression values were harmonized before dataset integration. Batch effects attributable to dataset origin were subsequently adjusted using ComBat, with disease status included as a biological covariate to preserve keloid-related expression differences. The integrated GSE90051–GSE158395 cohort was then used as an independent external validation dataset. ROC curves were constructed for VCAM1 and FGFR1, and the corresponding areas under the curve (AUC) were calculated. Differential expression analyses were also performed between keloid and control samples to assess whether the expression patterns identified in the training dataset could be independently reproduced.

### 2.6 Correlation analysis between candidate genes and ssGSEA-derived immune infiltration scores

To investigate the relationship between candidate genes and immune cell infiltration, we first extracted the expression levels of candidate genes from each sample. Using ssGSEA, we calculated enrichment scores for each immune feature, generating an immune infiltration score matrix. To quantify the correlation between immune infiltration and candidate gene expression, pairwise Spearman rank correlation tests were performed for each gene and immune feature score, calculating correlation coefficients and p-values. Significant associations were visualized through correlation heatmaps and scatter plots depicting key gene-immune feature relationships.

### 2.7 Molecular docking analysis and molecular dynamics simulations of candidate targets with pyrethroid-related compounds

To evaluate the binding affinity between the candidate genes and the six pyrethroid insecticide components, we first retrieved the SDF structures of the relevant proteins from the PDB database. Molecular docking was then performed utilizing the CB-Dock2 platform, where the 3D structures of the pyrethroid components were docked against the SDF structures of the candidate proteins. The docking results were evaluated by calculating the corresponding Vina scores to determine binding affinity.

Molecular dynamics (MD) simulations were performed using GROMACS 2022.2 to evaluate the stability and conformational dynamics of the protein–ligand complexes. Protein topology files were generated using the pdb2gmx module with the Amber99SB-ILDN force field. Ligand parameters were generated using the GAFF2 force field with sobtop_1.0 (dev3.1), and ligand atomic charges were assigned using the RESP method. Each complex was solvated in a cubic box using the TIP3P water model, with a minimum distance of 1.0 nm between the solute and the box boundary. Na⁺ and Cl⁻ ions were added using gmx genion to neutralize the system and achieve a physiological ionic strength of 0.15 M. Long-range electrostatic interactions were treated using the particle mesh Ewald (PME) method, with a 1.2 nm cutoff applied to van der Waals and short-range electrostatic interactions. Bonds involving hydrogen atoms were constrained using the LINCS algorithm, and a 2 fs integration time step was used. Energy minimization was performed using steepest descent and conjugate-gradient algorithms, followed by 1 ns NVT and 1 ns NPT equilibration with positional restraints. The temperature was maintained at 300 K using the V-rescale thermostat, and the pressure was maintained at 1 bar using the Parrinello–Rahman barostat. After equilibration, unrestrained production MD simulations were performed for 100 ns. Trajectory analyses were conducted using standard GROMACS tools, including RMSD (gmx rms) to assess structural stability, RMSF (gmx rmsf) to evaluate residue flexibility, radius of gyration (Rg; gmx gyrate) to assess structural compactness, solvent-accessible surface area (SASA; gmx sasa) to evaluate solvent exposure, and hydrogen-bond analysis (gmx hbond) to characterize intermolecular interactions. Principal component analysis (PCA) and free-energy landscape (FEL) analysis were additionally performed to characterize dominant conformational states and energy distributions.

### 2.8 Single-cell sequencing analysis

To explore the distribution and expression levels of candidate genes across keloid cell subtypes, we processed and quality-controlled the GSE163973 [23] single-cell dataset. Dimensionality reduction and clustering analyses were performed to identify distinct cell populations, followed by cell annotation. This approach enabled us to map the expression of the candidate genes within specific keloid cell subtypes.

### 2.9 Virtual gene knockout

To explore the potential downstream regulatory effects of VCAM1, we applied the scTenifoldKnk algorithm [24] to perform an in silico knockout of VCAM1 in endothelial cells derived from the GSE163973 single-cell RNA-seq dataset.

### 2.10 Experimental validation

To validate the bioinformatics findings and further assess the effect of cypermethrin exposure on VCAM1 expression in keloid-derived fibroblasts, human keloid-derived fibroblast-like KEL-FIB cells (ATCC CRL-1762) were cultured in DMEM supplemented with 10% fetal bovine serum (FBS) at 37°C in a humidified atmosphere containing 5% CO_2_. When the cells reached an appropriate confluence, they were divided into two groups: a vehicle control group and a cypermethrin-treated group. Cells in the treatment group were exposed to 20 μM cypermethrin for 24 h, whereas cells in the control group received an equivalent volume of vehicle. Total RNA was extracted using TRIzol reagent and reverse-transcribed into cDNA using the All-in-One RT Master Mix in a 20-μL reaction system. The synthesized cDNA was diluted with RNase-free water and subjected to quantitative real-time PCR (qRT-PCR) using SYBR Green qPCR Master Mix to determine VCAM1 mRNA expression. β-actin (ACTB) was used as the internal reference gene, and relative VCAM1 expression was calculated using the 2^ − ΔΔCt method. Each sample was analyzed in triplicate to ensure technical reproducibility. The primer sequences for VCAM1 and ACTB are listed in Table 1.

**Table 1:** Primer sequences of VCAM1 genes.

Prime name	Sequence
VCAM1-F	CCGTCTCATTGACTTGCAGC
VCAM1-R	GATGTGGTCCCCTCATTCGT

## 3 Results

### 3.1 Identification of gene targets associated with pyrethroid insecticides

A total of 623 genes associated with cyphenothrin were identified from three databases: SEA, SwissTargetPrediction, and TargetNet (Fig 1A). For dimefluthrin, 609 genes were retrieved from SwissTargetPrediction and TargetNet (Fig 1B). Similarly, 610 genes for imiprothrin were obtained from SwissTargetPrediction and TargetNet (Fig 1C), while 609 genes for prallethrin were gathered from the same two databases (Fig 1D). For theta-cypermethrin, 625 genes were extracted from SEA, SwissTargetPrediction, and TargetNet (Fig 1E), and 621 genes for transfluthrin were identified from SwissTargetPrediction and TargetNet (Fig 1F). Finally, the gene sets for all six pyrethroid insecticide components were deduplicated and combined, yielding 710 unique genes related to pyrethroid insecticides.

**Fig 1:**
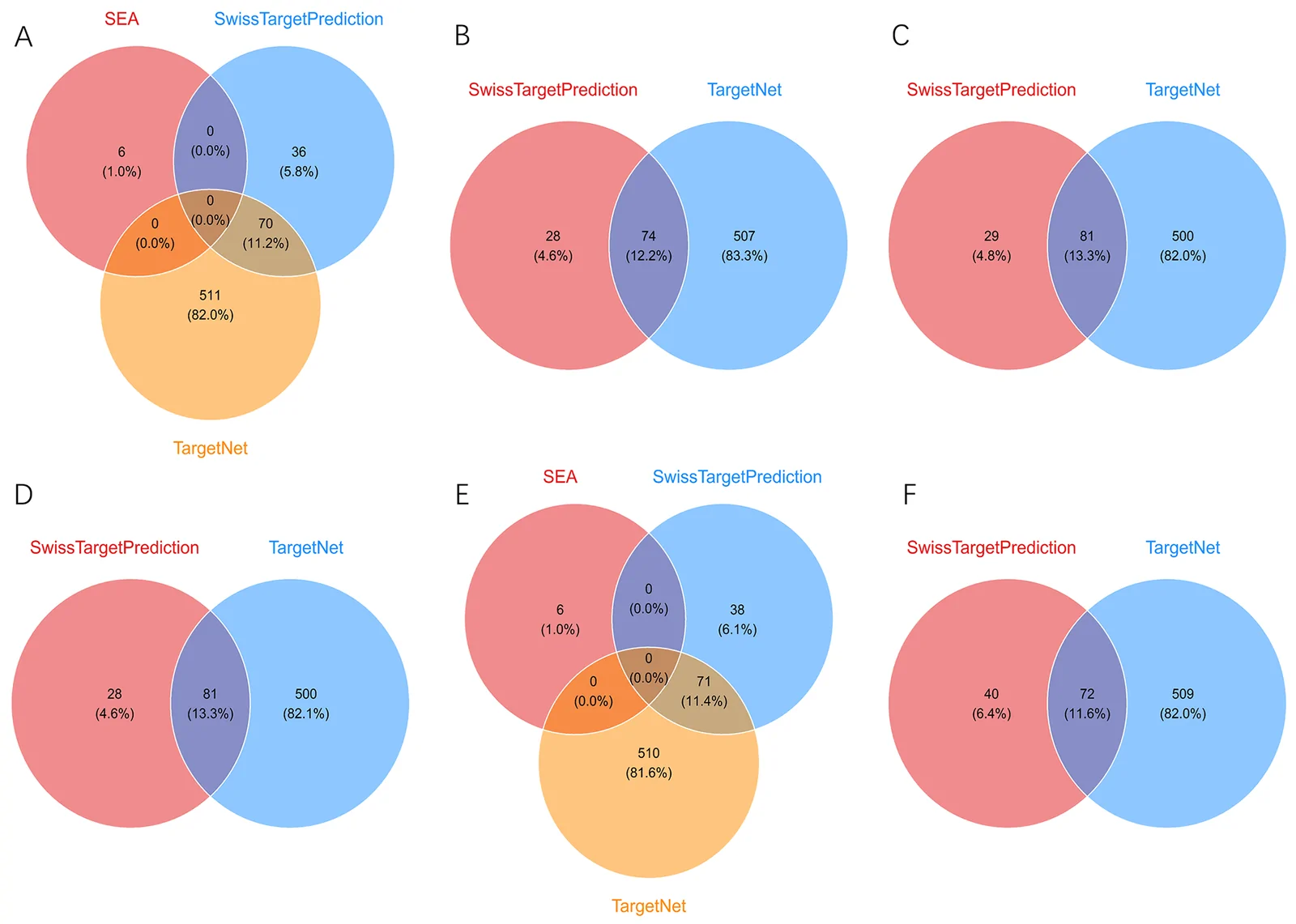
Identification of gene targets associated with pyrethroid insecticides. (A). Venn diagram illustrating Cyphenothrin-related target genes shared between SEA, TargetNet and SwissTargetPrediction databases. (B). Venn diagram illustrating Dimefluthrin-related target genes shared between TargetNet and SwissTargetPrediction databases. (C). Venn diagram illustrating Imiprothrin-related target genes shared between TargetNet and SwissTargetPrediction databases. (D). Venn diagram illustrating Prallethrin-related target genes shared between TargetNet and SwissTargetPrediction databases. (E). Venn diagram illustrating Theta-Cypermethrin-related target genes shared between SEA, TargetNet and SwissTargetPrediction databases. (F). Venn diagram illustrating Transfluthrin-related target genes shared between TargetNet and SwissTargetPrediction databases.

### 3.2 Identification of gene targets associated with Keloids

We performed differential expression analysis using the GSE145725 dataset, identifying 376 differentially expressed genes (DEGs) associated with keloids (Fig 2A-2C). Additionally, 836, 4, and 5 keloid-related genes were obtained from the TTD, OMIM, and GeneCards databases, respectively. After deduplication and integration, we compiled a total of 1187 unique keloid-related gene targets (Fig 2D). By intersecting the pyrethroid insecticide-related genes with the keloid-related genes, we identified 127 common genes shared between pyrethroid insecticides and keloids (Fig 2E).

**Fig 2:**
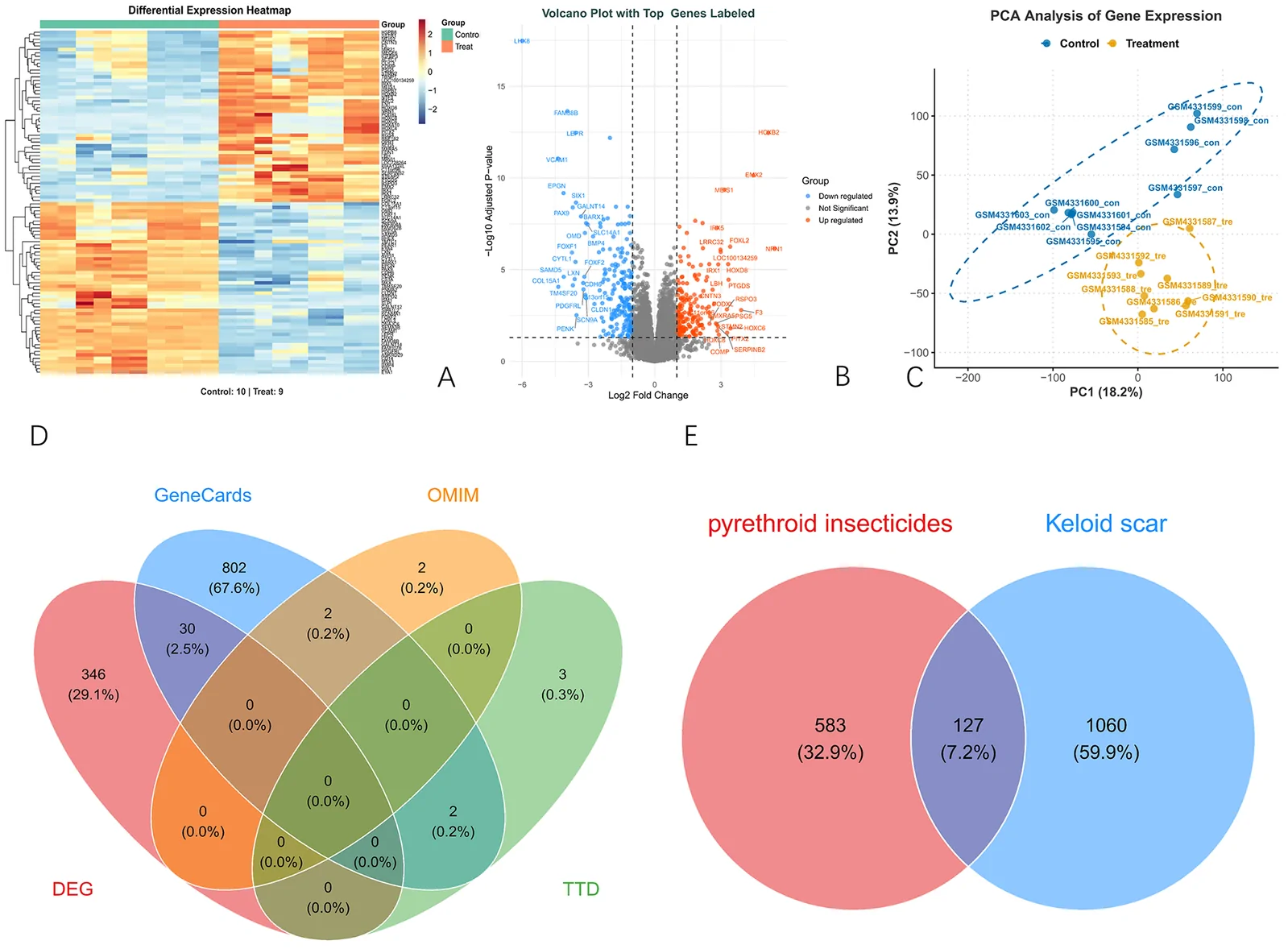
Identification of gene targets associated with pyrethroid-keloid. (A). Heatmap of differentially expressed genes in GSE145725. (B). Volcano diagram of differentially expressed genes in GSE145725. (C). PCA diagram of differentially expressed genes in GSE145725. (D). Venn diagram illustrating keloid-related target genes shared between DEGs, GeneCards, OMIM and TTD databases. (E). Venn diagram illustrating pyrethroid-keloid-related target genes.

### 3.3 Functional enrichment analysis and PPI network construction for pyrethroid-keloid co-genes

GO enrichment analysis disclosured that the 127 common genes between pyrethroid insecticides and keloids are significantly involved in the positive regulation of biological processes such as the MAPK cascade, membrane raft formation, and endopeptidase activity (Fig 3A). KEGG enrichment analysis indicated that these genes are enriched in key signaling pathways, including the HIF–1, MAPK, PI3K–Akt, and TNF signaling pathways (Fig 3B). The constructed PPI network further highlighted the significant interactions among these 127 genes (Fig 3C).

**Fig 3:**
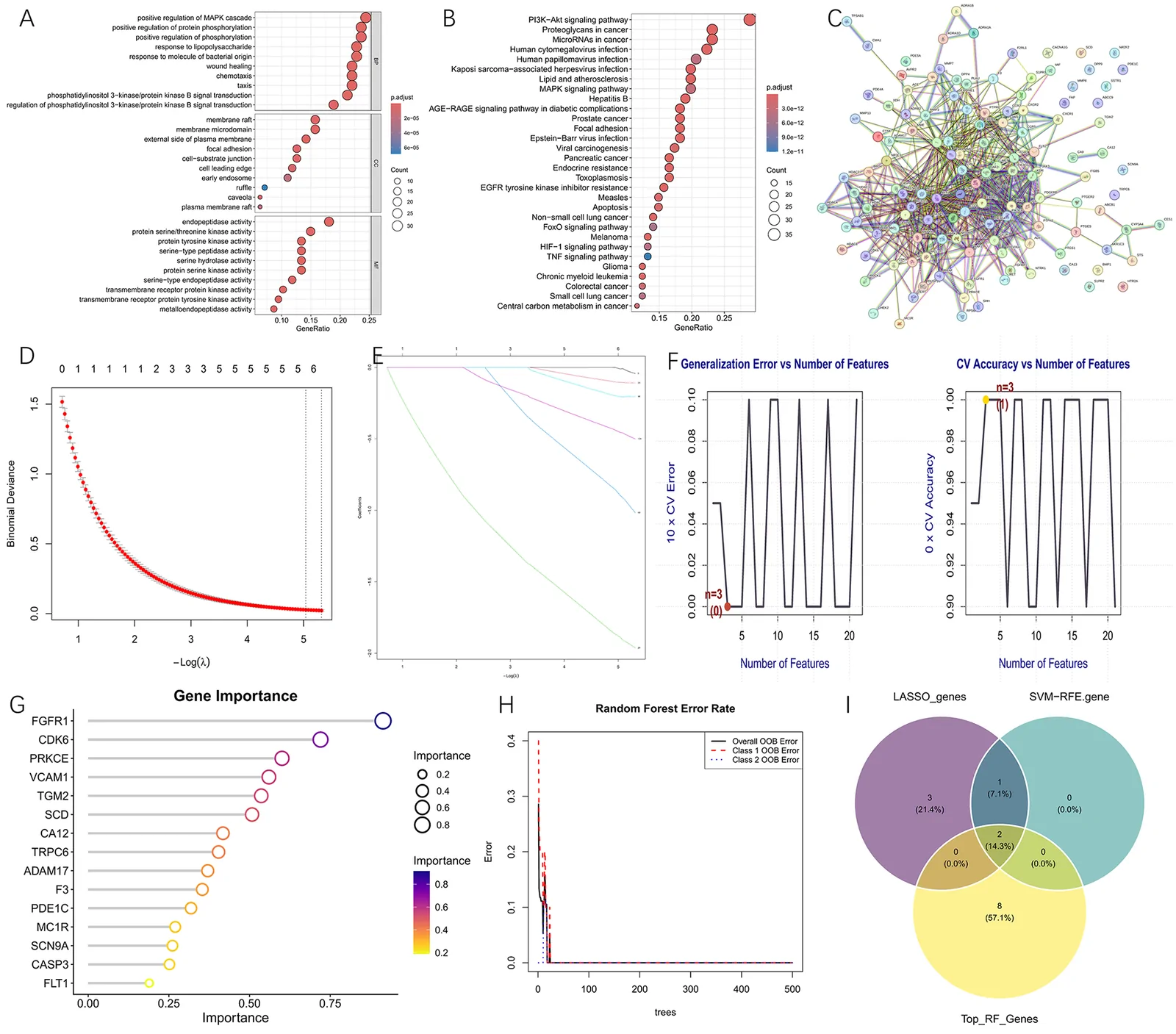
(A) Represent the GO enrichment analysis of pyrethroid-keloid targets. (B) Represented KEGG enrichment analysis of pyrethroid-keloid targets. (C) PPI network of pyrethroid-keloid-related targets constructed using STRING database. (D-E). Lasso regression identified 6 core diagnostic genes. (F). SVM-RFE identified 3 core diagnostic genes. (G-H). The randomized tree identified 10 core diagnostic genes. (I). Three machine learning methods identified two core targets of pyrethroid insecticides- keloid.

### 3.4 Machine learning-based identification of core components in pyrethroid-keloid interaction

This study used three machine learning methods to further screen candidate core genes of pyrethroid-keloid. First, lasso regression identified 6 characteristic genes (Fig 3D-3E). Second, SVM-RFE identified 3 characteristic genes (Fig 3F); subsequently, randomized tree analysis identified 10 characteristic genes (Fig 3G-3H). Eventually, the intersection of the characteristic genes identified by the three machine learning methods yielded two candidate genes, namely VCAM1 and FGFR1 (Fig 3I).

### 3.5 Diagnostic efficacy of core candidate genes in keloids

In the training dataset GSE145725, VCAM1 and FGFR1 showed statistically significant differential expression between keloid and control samples and demonstrated excellent dataset-level discriminatory performance, with AUC values of 1.000 for both genes. In the integrated external validation cohort generated from GSE90051 and GSE158395, VCAM1 retained good discriminatory performance, with an AUC of 0.869, whereas FGFR1 showed more limited discriminatory ability, with an AUC of 0.677 (Fig 4A). Differential expression analysis further showed that VCAM1 was significantly upregulated in keloid samples compared with controls, whereas FGFR1 did not show a statistically significant between-group difference (Fig 4B). These findings provide additional external support for VCAM1 as a prioritized candidate gene associated with keloid, while the evidence for FGFR1 was less consistent and should therefore be interpreted more cautiously.

**Fig 4:**
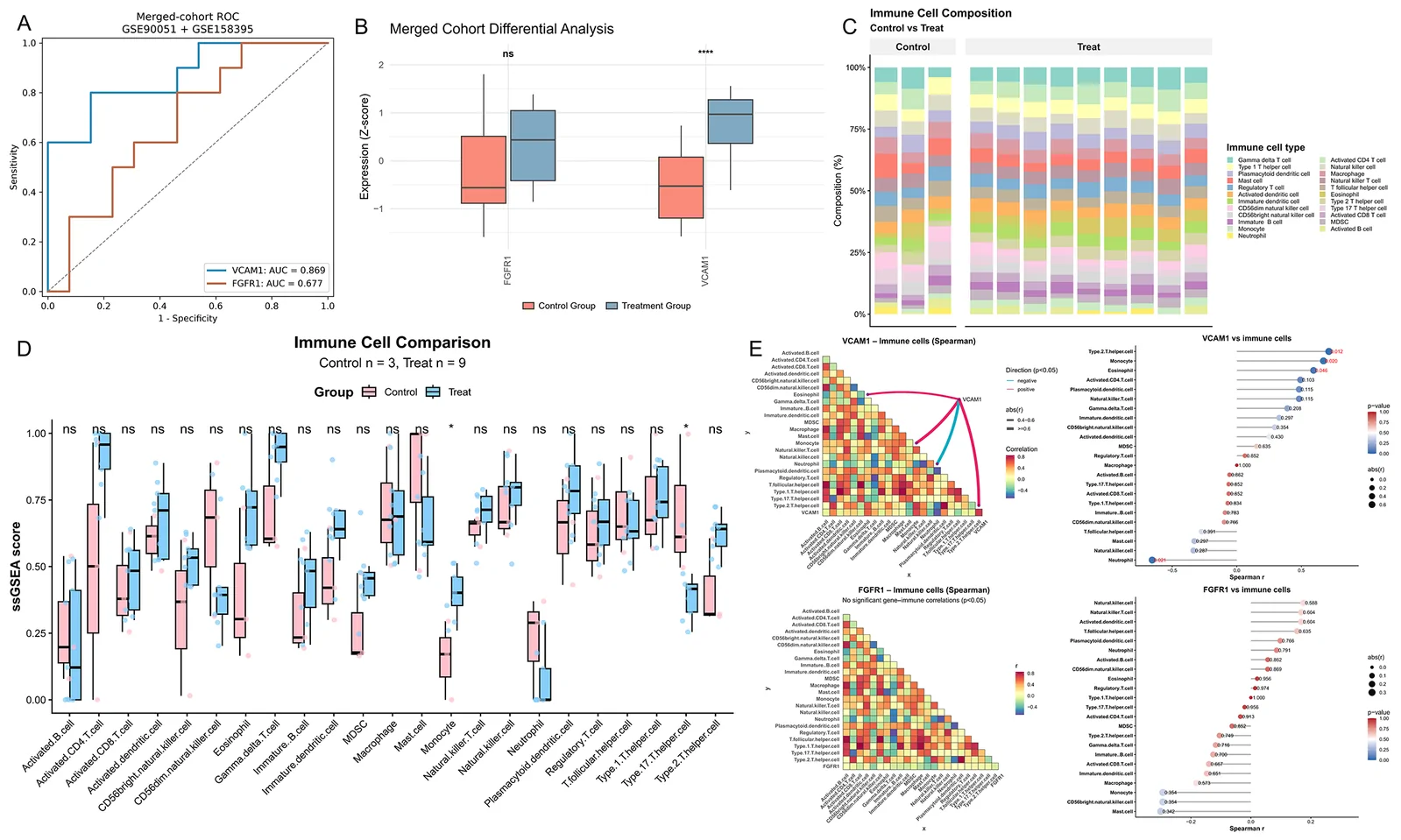
External validation and immune landscape associated with VCAM1 and FGFR1. (A) ROC analysis of VCAM1 and FGFR1 in the integrated GSE90051–GSE158395 validation cohort. (B) Differential expression of VCAM1 and FGFR1 between keloid and control samples in the same cohort. (C–D) Immune infiltration analysis using GSE145725. (E) Correlations between VCAM1/FGFR1 expression and 22 immune cell types.

### 3.6 Correlation between candidate genes and immune cells

First, we processed the GSE145725 dataset to generate a standardized expression matrix and used the ssGSEA method to calculate the enrichment scores for each sample across immune gene sets, resulting in an immune infiltration score matrix (Fig 4C-4D). Subsequently, we calculated the correlation between VCAM1 and FGFR1 expression levels and various immune cell types (Fig 4E). Notably, VCAM1 expression was negatively correlated with neutrophils and positively correlated with eosinophils, monocytes, and type 2 helper T cells.

### 3.7 Molecular docking analysis and MD simulations of candidate genes and pyrethroid insecticides

Molecular docking yielded the following binding energies: cyphenothrin with FGFR1 and VCAM1, −8.8 and −8.4 kcal/mol, respectively; dimefluthrin, −8.1 and −8.2 kcal/mol; imiprothrin, −7.4 and −7.2 kcal/mol; prallethrin, −8.2 and −7.8 kcal/mol; theta-cypermethrin, −8.8 kcal/mol for both FGFR1 and VCAM1; and transfluthrin, −8.3 and −8.0 kcal/mol, respectively. These docking scores suggest energetically favorable predicted interactions between the six pyrethroid compounds and the two candidate proteins (Fig 5).

**Fig 5:**
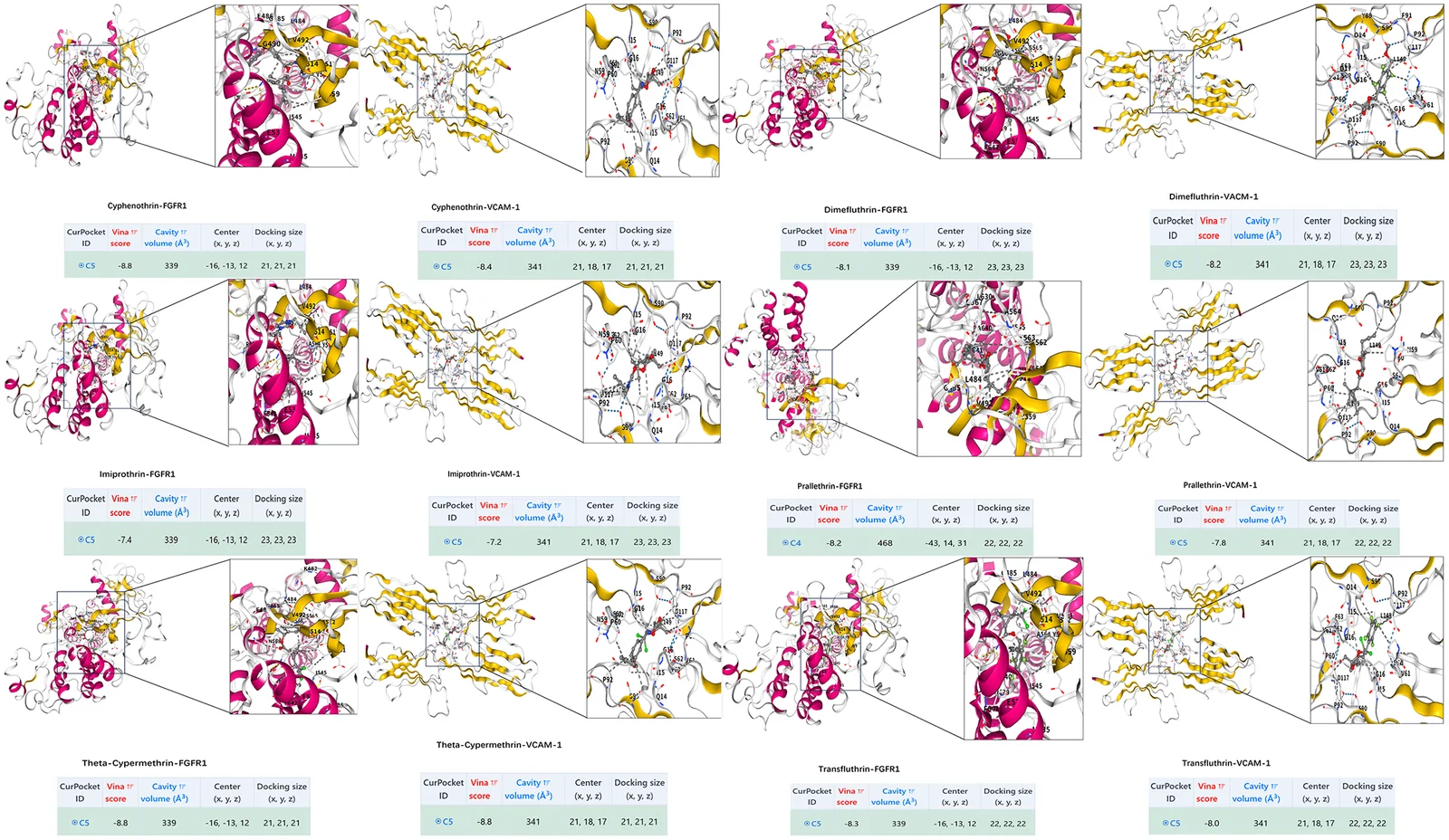
Molecular docking of 2 core genes with pyrethroid insecticides.

To further assess the conformational behavior of the VCAM1–theta-cypermethrin complex, a 100-ns molecular dynamics simulation was performed based on the docking pose. RMSD analysis showed that VCAM1 and the VCAM1–theta-cypermethrin complex reached relatively stable trajectories after approximately 45 ns, with average RMSD values of approximately 0.28 nm and 0.23 nm, respectively (S1A Fig). The comparable RMSD profiles suggested that theta-cypermethrin binding was not associated with major global conformational rearrangements of VCAM1 during the simulated period. Rg analysis indicated that the complex maintained relatively stable structural compactness throughout the trajectory (S1B Fig), while SASA showed only limited fluctuations, suggesting no marked change in solvent exposure (S1C Fig). Hydrogen-bond analysis showed 0–3 intermolecular hydrogen bonds during the simulation, with approximately one hydrogen bond present in most frames (S1D Fig). RMSF analysis further showed that most residues fluctuated by less than 0.3 nm, indicating limited local flexibility across much of the complex (S1E Fig). FEL analysis identified a predominant low-energy conformational basin centered approximately at PC1 = 0.17 and PC2 = 2.42 (S1F Fig). Collectively, these results provide supportive computational evidence that the docked VCAM1–theta-cypermethrin complex can maintain a relatively stable conformation over the 100-ns simulation period. However, these findings should not be interpreted as direct experimental evidence of binding affinity or biological interaction.

### 3.8 Expression levels and distribution of candidate genes in keloid cell subtypes

The GSE163973 dataset was annotated into 11 distinct cell subtypes: lymphatic endothelial cells, keratinocytes, smooth muscle cells, fibroblasts, mast cells, cycling cells, myeloid cells, neural cells, endothelial cells, T cells, and sweat gland cells (Fig 6A). FGFR1 was principally expressed in endothelial and mast cells, with relatively high expression also observed in fibroblasts, smooth muscle cells, keratinocytes, and myeloid cells. VCAM1 indicated high expression in endothelial and mast cells, although at lower levels than FGFR1, and was also expressed at notable levels in fibroblasts and smooth muscle cells (Fig 6B-6D).

**Fig 6:**
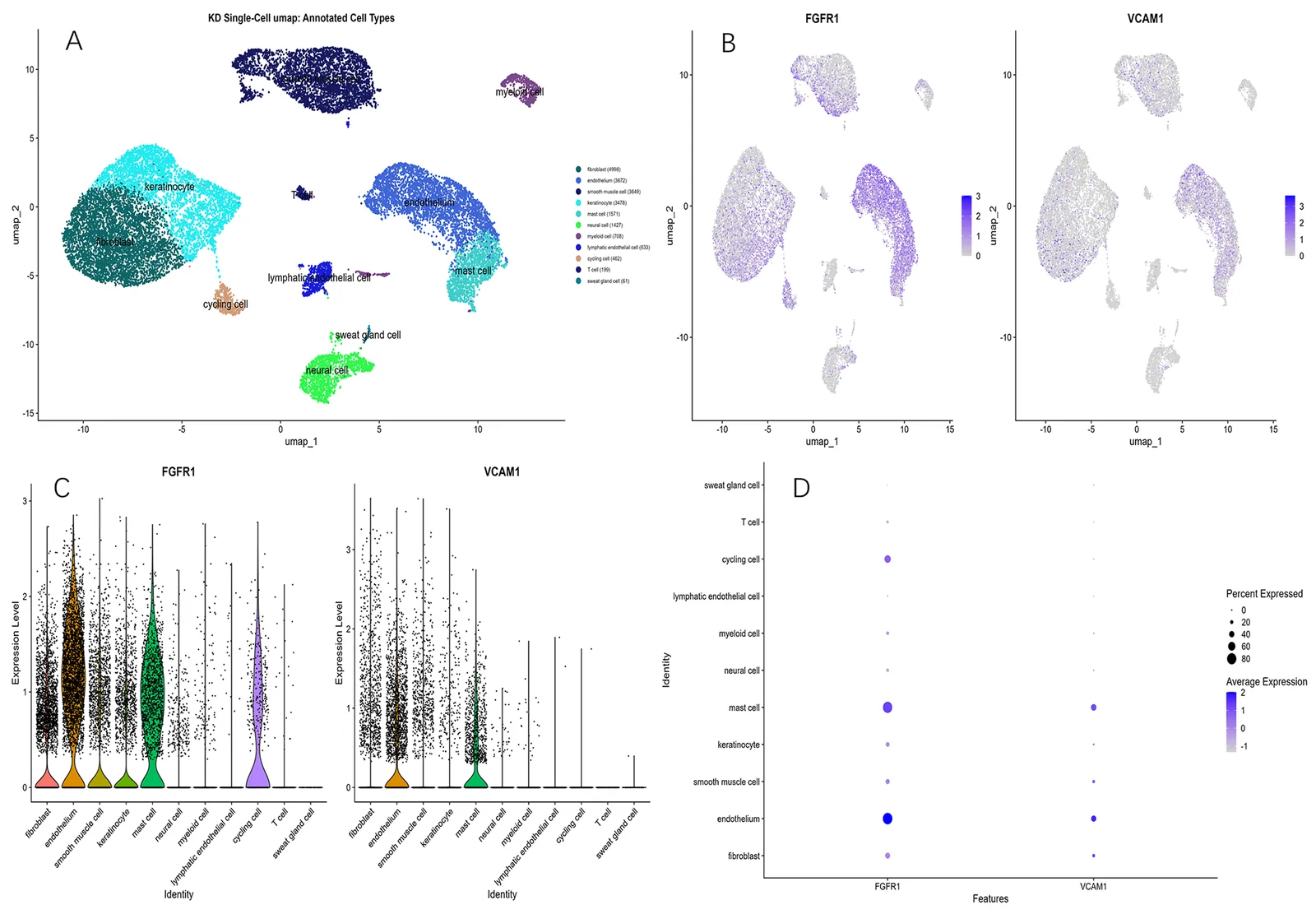
The expression levels of 2 core genes in keloid cell subtypes.

### 3.9 Virtual gene perturbation suggests a potential VCAM1–SOCS3 regulatory relationship in keloid

In the GSE163973 single-cell dataset, in silico knockout of VCAM1 in endothelial cells using the scTenifoldKnk algorithm identified SOCS3 as the most significantly perturbed downstream gene. These findings suggest a potential regulatory relationship between VCAM1 and SOCS3 in endothelial cells and nominate the VCAM1–SOCS3 association as a hypothesis-generating pathway that may be relevant to pyrethroid-associated keloid alterations (Fig 7). However, this relationship is based on computational perturbation analysis and requires direct experimental validation.

**Fig 7:**
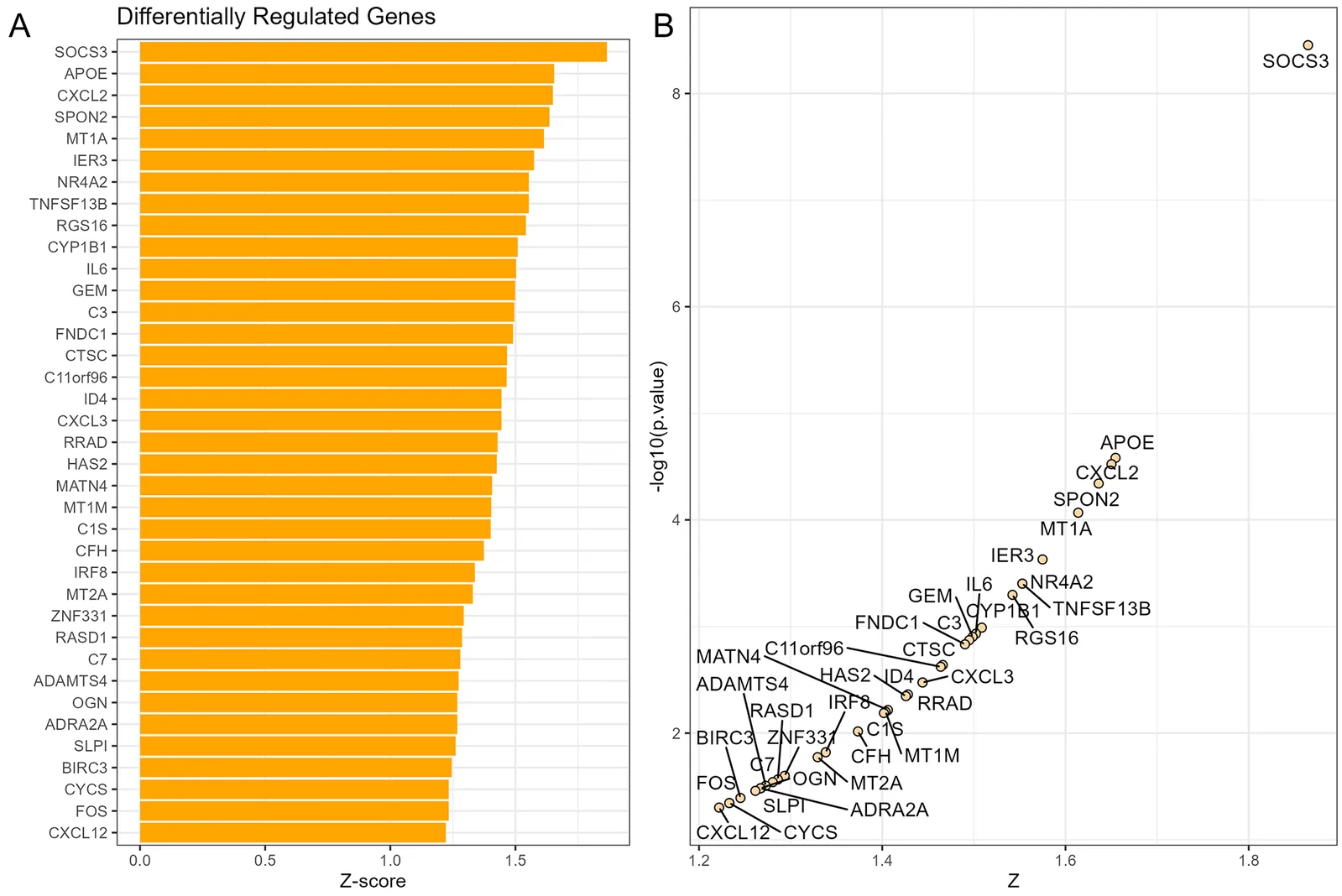
Virtual knockout reveals the underlying mechanisms by which VCAM1 promote keloid.

### 3.10 Validation of key target expression

qRT-PCR analysis showed that VCAM1 mRNA expression was significantly increased in KEL FIB cells following 24 h exposure to 20 μM cypermethrin compared with the vehicle control group. The relative expression level of VCAM1 increased from approximately 1.0 in untreated KEL FIB cells to about 2.2 after cypermethrin treatment (P < 0.0001; Fig 8), indicating that cypermethrin exposure markedly upregulated VCAM1 expression in keloid-derived fibroblasts.

**Fig 8:**
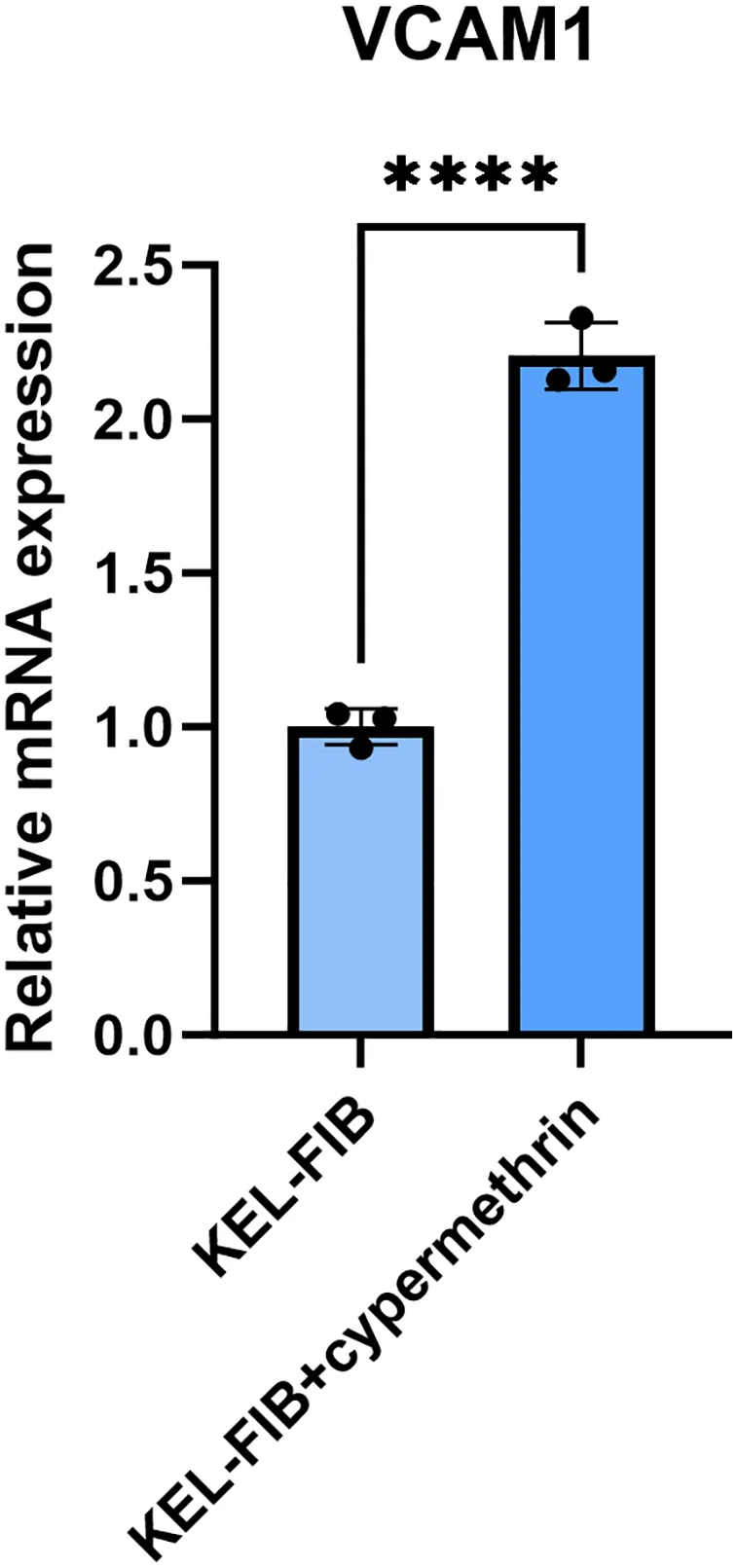
Experimental validation of VCAM1 expression in keloid-derived fibroblasts. Relative VCAM1 mRNA expression was determined by qRT-PCR in normal human adult dermal fibroblasts (HDFa) and keloid-derived KEL-FIB cells. β-actin was used as the internal reference gene, and relative expression was calculated using the 2^ − ΔΔCt method. Data are presented as mean ± SD from three independent measurements. ****P < 0.0001.

## 4 Discussions

Pyrethroid insecticides are extensively used in agriculture and household products globally. Previous studies have highlighted their developmental toxicity, with significant adverse effects on children’s cognitive and behavioral development, both in utero and during early childhood [8]. In addition, pyrethroid insecticides exhibit significant neurotoxicity [25]. Specifically, pyrethroid-related bisphenol A has been shown to induce neuronal damage, neuroinflammation, and oxidative stress, leading to memory and cognitive impairments in the hippocampus of mice [26]. In the present study, network toxicology combined with machine-learning approaches prioritized VCAM1 and FGFR1 as candidate molecular targets potentially linking pyrethroid exposure with keloid-associated alterations. External transcriptomic validation provided stronger support for VCAM1 than for FGFR1, while molecular docking and 100-ns molecular dynamics simulations provided supportive computational evidence for selected pyrethroid–target interactions. Single-cell transcriptomic analysis further characterized the cell-type-specific expression and distribution of the candidate genes, and immune infiltration analysis identified associations between VCAM1 expression and several immune-cell populations. Importantly, qRT-PCR experiments showed that exposure of KEL FIB cells to 20 μM cypermethrin for 24 h significantly increased VCAM1 mRNA expression, providing experimental expression-level support for VCAM1 as a cypermethrin-responsive candidate gene. Virtual gene perturbation further suggested a putative regulatory relationship between VCAM1 and SOCS3. Collectively, these findings support VCAM1 as a prioritized candidate potentially involved in pyrethroid-associated molecular alterations in keloid, whereas the predicted VCAM1–SOCS3 relationship remains hypothesis-generating and requires direct mechanistic validation.

Keloid formation is a complex process characterized by abnormal fibroblast proliferation, excessive extracellular matrix accumulation, immune response activation, and dysregulated signaling pathways [27,28]. The overactivation of pathways such as TGF-β, Wnt, Notch, and JAK/STAT, in conjunction with local immune responses, genetic mutations, and epigenetic modifications, leads to uncontrolled fibrosis, which ultimately results in keloid formation [29]. Environmental toxins can contribute to the progression and onset of keloids via various mechanisms, including direct skin damage, immune response activation, enhancement of oxidative stress, and modulation of gene expression. Long-term exposure to environmental toxins such as heavy metals, air pollution, and chemicals can exacerbate fibrosis and promote keloid formation, particularly when the skin is already damaged [30]. This study is the first to elucidate how pyrethroid insecticides contribute to keloid formation, with inflammation as a key mechanism. Relevant signaling pathways implicated in this process include PI3K–Akt, MAPK, HIF–1, and TNF signaling pathways.

This study identified VCAM1 and FGFR1 as candidate molecular targets potentially associated with pyrethroid-related keloid alterations, with stronger external and experimental support for VCAM1. FGFR1 is a receptor tyrosine kinase involved in different physiological processes, such as cell angiogenesis, differentiation, proliferation, migration, and neural development. It activates downstream signaling pathways by binding to its ligand, fibroblast growth factor (FGF). FGFR1 plays a pivotal role in multiple tissues and organs, especially in pathological conditions like tumors and fibrosis [31,32]. Research has shown that FGFR1 can inhibit the bioactivity of keloid fibroblasts via the FGFR signaling pathway [33]. Single-cell sequencing further revealed high expression of FGFR1 in fibroblasts and mast cells, suggesting that FGFR1 may contribute to fibrosis through fibroblasts and modulate inflammation via related immune cells, thereby promoting keloid formation. VCAM1 is a transmembrane immunoglobulin primarily expressed on endothelial cells, where it plays a critical role in the adhesion and migration of leukocytes to vascular endothelial cells. It is a key molecule facilitating immune cell penetration into inflammatory sites and is deeply involved in immune responses and inflammation [34]. Previous studies integrating single-cell and bulk transcriptomic analyses have shown that VCAM1 is highly expressed in keloids and is strongly associated with immune modulation, further implicating it in the pathogenesis of keloid formation [35,36]. Immune infiltration analysis revealed associations between VCAM1 expression and several immune cell populations, while single-cell transcriptomic analysis showed relatively high VCAM1 expression in endothelial cells and mast cells. These findings suggest a potential involvement of VCAM1 in the immune microenvironment of keloid, although its specific immunoregulatory role requires further experimental validation.

Although this study integrated multi-omics, computational analyses, external transcriptomic validation, and qRT-PCR experiments to investigate potential molecular links between pyrethroid exposure and keloid-associated alterations, several limitations should be acknowledged. First, the mechanistic evidence remains largely computational. Although qRT-PCR showed that cypermethrin exposure increased VCAM1 mRNA expression in KEL FIB cells, this finding provides only expression-level support and does not establish a causal VCAM1–SOCS3 regulatory mechanism. Second, the predicted VCAM1–SOCS3 relationship was inferred from virtual gene perturbation and has not been validated by genetic knockdown, overexpression, rescue experiments, or direct protein-level analyses. Third, the molecular docking and 100-ns molecular dynamics simulations provide supportive structural evidence but do not demonstrate direct biochemical binding or in vivo target engagement. Finally, the present study did not fully address exposure-related factors such as dose relevance, route of exposure, metabolism, tissue distribution, and bioavailability. Future studies incorporating mechanistic cell experiments, direct binding assays, and appropriate in vivo models are required to validate the biological significance of the proposed VCAM1-associated pathway in pyrethroid-related keloid alterations.

## 5 Conclusion

In summary, this study integrated network toxicology, machine learning, external transcriptomic validation, molecular docking, molecular dynamics simulation, single-cell transcriptomics, virtual gene perturbation, and qRT-PCR to investigate potential molecular links between pyrethroid exposure and keloid-associated alterations. VCAM1 and FGFR1 were prioritized as candidate genes, with stronger external and experimental support for VCAM1. In the integrated validation cohort, VCAM1 showed good discriminatory performance (AUC = 0.869) and was significantly upregulated in keloid samples. Molecular docking and 100-ns molecular dynamics simulation provided supportive computational evidence for selected pyrethroid–target interactions, while qRT-PCR showed that cypermethrin exposure increased VCAM1 expression in KEL FIB cells. Virtual gene perturbation further suggested a putative VCAM1–SOCS3 regulatory relationship. Collectively, these findings support VCAM1 as a prioritized candidate in pyrethroid-associated keloid alterations, although the proposed VCAM1–SOCS3 relationship requires further mechanistic validation.

## Supporting information

S1 Fig Molecular dynamics simulations of VCAM1 and Theta-Cypermethrin. (A) Root-mean-square deviation (RMSD) profiles of the VCAM1–θ-cypermethrin complex, θ-cypermethrin, and VCAM1 during the 100-ns MD simulation. (B) Radius of gyration (Rg) of the VCAM1–θ-cypermethrin complex over the simulation period, reflecting its overall structural compactness. (C) Solvent-accessible surface area (SASA) of the complex throughout the simulation. (D) Number of intermolecular hydrogen bonds formed during the simulation. (E) Root-mean-square fluctuation (RMSF) of individual VCAM1 residues, indicating residue-level flexibility. (F, G) Two-dimensional and three-dimensional Gibbs free energy landscapes of the VCAM1–θ-cypermethrin complex projected onto the first two principal components (PC1 and PC2). The color scale represents the relative Gibbs free energy, with lower-energy regions indicating more thermodynamically favorable conformational states.
(DOCX)

S1 File Supplementary datasets containing the predicted pyrethroid-related targets, keloid-associated genes, shared genes between pyrethroids and keloids, and candidate genes identified through machine learning approaches, including LASSO regression, SVM-RFE, and random forest. The file also includes the overlapping genes identified by the three machine learning algorithms.
(XLS)

## Glossary

ROC: receiver operating characteristic
PPI: protein–protein interaction
KEGG: Kyoto Encyclopedia of Genes and Genomes
GO: Gene Ontology
SVM-RFE: support vector machine–recursive feature elimination
AUC: area under the curve
DEGs: differentially expressed genes
MD: Molecular dynamics
PME: Particle Mesh Ewald
PCA: Principal component analysis
FEL: free energy landscape
