# Supplementary material for: Integrated network toxicology and experimental validation identify VCAM1 as a candidate molecular link between pyrethroid exposure and keloid
Source: PLoS One. 2026 Nov 10;21(11):e0362474. doi: 10.1371/journal.pone.0362474 (PMC13750557; doi:10.1371/journal.pone.0362474)
Supplement: Supplementary file 1 [file pone.0362474.s001.docx]

Fig.S1：Molecular dynamics simulations of VCAM1 and Theta-Cypermethrin.

(A) Root-mean-square deviation (RMSD) profiles of the VCAM1–θ-cypermethrin complex, θ-cypermethrin, and VCAM1 during the 100-ns MD simulation. (B) Radius of gyration (Rg) of the VCAM1–θ-cypermethrin complex over the simulation period, reflecting its overall structural compactness. (C) Solvent-accessible surface area (SASA) of the complex throughout the simulation. (D) Number of intermolecular hydrogen bonds formed during the simulation. (E) Root-mean-square fluctuation (RMSF) of individual VCAM1 residues, indicating residue-level flexibility. (F, G) Two-dimensional and three-dimensional Gibbs free energy landscapes of the VCAM1–θ-cypermethrin complex projected onto the first two principal components (PC1 and PC2). The color scale represents the relative Gibbs free energy, with lower-energy regions indicating more thermodynamically favorable conformational states.
